# Improving equity in Alzheimer's disease research: Biomarker disclosure in underserved populations

**DOI:** 10.1002/alz70856_107224

**Published:** 2026-01-08

**Authors:** Diana C Oviedo, Rasheda Haughbrook, Carli Culjat, Alcibiades E Villarreal, Ladanya Ramirez, Maria B Carreira, Oriana Batista, Sherelle Harmon, Zhuo Meng, Adam E. Tratner, Casey Xavier Hall, Eugenia Millender, Gabrielle B Britton

**Affiliations:** ^1^ Sistema Nacional de Investigación (SNI), Panamá, Panamá, Panama; ^2^ Universidad Santa María La Antigua, Panamá, Panamá, Panama; ^3^ Florida State University, Tallahassee, FL, USA; ^4^ Instituto de Investigaciones Científicas y Servicios de Alta Tecnología (INDICASAT AIP), Panama, Panama; ^5^ Florida State University, Panama, Panama, Panama; ^6^ Instituto de Investigaciones Científicas y Servicios de Alta Tecnología (INDICASAT AIP), Centro de Neurociencias, Panamá, Panamá, Panama; ^7^ Universidad Autónoma de Chiriquí, David, Chiriquí, Panama; ^8^ Escuela de Psicología, Universidad Santa María la Antigua (USMA), Panamá, Panamá, Panama

## Abstract

**Background:**

The early diagnosis of Alzheimer's disease (AD) demands reliable biomarkers and accessible detection methods. Evidence shows that combining brain imaging with cerebrospinal fluid (CSF) biomarkers significantly enhances the sensitivity and specificity of AD diagnosis. As biomarkers are included in diagnostic criteria, disclosing biomarker results to participants becomes crucial. However, in low‐resource settings—where healthcare access, provider training, and patient support are often limited—disclosing AD biomarkers poses distinct ethical, logistical, and psychological challenges.

**Method:**

Informing research participants about their individual test results is a critical ethical obligation grounded in the principles of respect for persons, beneficence, and justice. Effective disclosure must also consider culture, individual circumstances, and varying levels of health and research literacy. To date, no studies in Latin America, to our knowledge, have incorporated biomarker disclosure in aging and dementia studies. In Panama, the Panama Aging Research Initiative – Health Disparities (PARI‐HD) program is addressing this gap by conducting a study to evaluate the diagnostic and prognostic value of biomarkers in blood, cerebrospinal fluid, and magnetic resonance imaging (MRI).

**Results:**

This study includes participants without cognitive impairment, individuals with mild cognitive impairment, AD, and Parkinson's disease. All participants provide informed consent and undergo a clinical evaluation, collection of information regarding health and functional status, cognitive testing, blood sampling to analyze serological and genetic biomarkers, lumbar puncture to obtain CSF, and brain structure evaluation using MRI. We propose to conduct a pilot research study where participants will receive biomarker results from a member of the research team that has previously received training in disclosure. Following disclosure, participants will receive a short questionnaire to assess comprehension of results and questionnaires to assess depression and anxiety symptoms. Psychological counseling will be available if requested and to individuals presenting mental health symptoms. Follow‐up calls will be conducted to assess individuals’ response to disclosure.

**Conclusion:**

This study will focus on context‐specific approach to AD biomarker disclosure that prioritizes patient well‐being in low‐resourced environments.

